# Potential Clinical Applications for Human Pluripotent Stem Cell-Derived Blood Components

**DOI:** 10.4061/2011/273076

**Published:** 2011-03-08

**Authors:** Erin A. Kimbrel, Shi-Jiang Lu

**Affiliations:** Stem Cell & Regenerative Medicine International, 33 Locke Drive, Marlborough, MA 01752, USA

## Abstract

The ability of human embryonic stem cells (hESCs) and induced pluripotent stem cells (iPSCs) to divide indefinitely without losing pluripotency and to theoretically differentiate into any cell type in the body makes them highly attractive cell sources for large scale regenerative medicine purposes. The current use of adult stem cell-derived products in hematologic intervention sets an important precedent and provides a guide for developing hESC/iPSC based therapies for the blood system. In this review, we highlight biological functions of mature cells of the blood, clinical conditions requiring the transfusion or stimulation of these cells, and the potential for hESC/iPSC-derivatives to serve as functional replacements. Many researchers have already been able to differentiate hESCs and/or iPSCs into specific mature blood cell types. For example, hESC-derived red blood cells and platelets are functional in tasks such as oxygen delivery and blood clotting, respectively and may be able to serve as substitutes for their donor-derived counterparts in emergencies. hESC-derived dendritic cells are functional in antigen-presentation and may be used as off-the-shelf vaccine therapies to stimulate antigen-specific immune responses against cancer cells. However, *in vitro* differentiation systems used to generate these cells will need further optimization before hESC/iPSC-derived blood components can be used clinically.

## 1. Introduction

Human embryonic stem cells (hESCs) have been touted as the future of regenerative medicine due to their potential to differentiate into any cell type in the body. Unlike adult or cord blood stem cells, hESCs are capable of expanding indefinitely in culture without losing their pluripotency, and this makes them an attractive cell source to be used for the large-scale production of a variety of therapeutic cell types [1]. The advent of human-induced pluripotent stem cells (iPSCs) has added another dimension to the field of regenerative medicine as it may allow patient-specific therapies to be produced, thus reducing issues with HLA mismatching and immunoincompatibility [2]. While each has its own advantages and disadvantages, hESCs and iPSCs represent two pluripotent cell sources with far-reaching clinical potential in treating neurologic disorders, repairing or replacing damaged tissues, and as detailed here, producing transfusable blood components.

**Figure 1:**
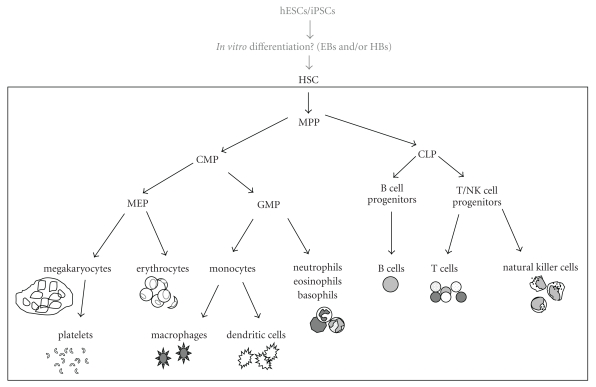
Simplified schematic of hematopoietic differentiation. At the top, hESCs and/or iPSCs may be able to recapitulate hematopoietic differentiation *in vitro* after initial differentiation into EBs and/or HBs intermediates. These culture-based intermediates differentiate into cells similar to mesoderm-derived HSC/progenitors. The boxed region shows hematopoietic differentiation as it is thought to occur *in vivo*. HSCs undergo successive stages of differentiation to give rise to progenitor cells in both the myeloid lineage (left side) and lymphoid lineage (right side). These progenitors will undergo further differentiation to eventually give rise to mature cells within the peripheral blood.

Hematopoietic stem cells (HSCs) located within the bone marrow normally give rise to and are responsible for replenishing all mature cells within the adult blood system [3]. HSCs initially differentiate into multipotent progenitors (MPPs) and then differentiate further into common myeloid progenitors (CMPs) and common lymphoid progenitors (CLPs). CMPs eventually give rise to erythrocytes, megakaryocytes/platelets, monocytes, and granulocytes while CLPs produce natural killer, T, and B cells (Figure 1). Researchers have already been able to obtain highly enriched populations of *in vitro* generated blood components by differentiating hESCs and/or iPSCs down particular hematopoietic lineages. Each of the mature cell types within the blood system can be used for distinct clinical purposes, and this paper will focus on the ability of hESCs/iPSCs to serve as substitutes for primary cells in these endeavors.

## 2. Red Blood Cells

**Figure 2:**
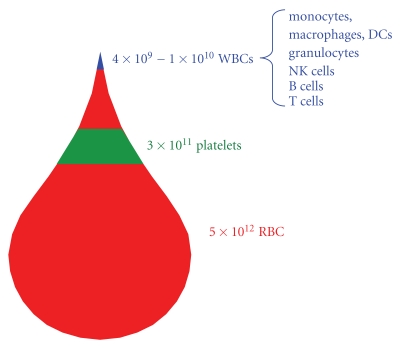
Number and type of cellular blood components per liter of human peripheral blood. hESCs and/or iPSCs may be able to serve as cost-effective, readily available substitutes for these various components of the peripheral blood. Both RBCs and platelets are frequently used in transfusions, but these donor-derived PB components are often in short supply. WBCs represent a very small percentage of PB cells, yet they serve critical functions in protecting the body from various microbes and cancer cells. They may be used in future cell-based therapies against cancer or HIV.

Erythrocytes or red blood cells (RBCs) are the most plentiful cell type in the peripheral blood and are present at a concentration of 5 × 10^12^ cells/liter(L) [4], which accounts for approximately 40%–45% of the total blood volume (Figure 2) [4, 5]. Despite the body's seemingly abundant capacity to produce RBCs, approximately 16 million units of RBCs are collected and transfused annually into patients [6], including those suffering from anemia (low RBC counts) or massive blood loss due to trauma. Type (O)Rh-negative “universal” RBCs are highly desirable for emergency situation where blood typing may not be possible and are usually the first to be depleted when clinics encounter shortages in their supplies. The derivation of (O)Rh-negative RBCs from hESCs/iPSCs clearly offers an attractive option for alleviating the constant shortage in donated RBCs.

Definitive erythropoiesis in the adult bone marrow is a multistep process regulated by the cytokine, erythropoietin (EPO). It begins when an HSC-derived CMP passes through the megakaryocyte erythrocyte progenitor (MEP) stage and commits to the erythroid lineage. The appearance of the pronormoblast (also called proerythroblast or rubriblast) marks the first stage of differentiation and is subsequently followed by early, intermediate, and late normoblast (erythroblast) stages, at which time the nucleus is expelled and the cell becomes a reticulocyte. Reticulocytes exit the bone marrow and become fully mature RBCs within the circulation, expressing adult forms of hemoglobin (*α*2*β*2) and delivering oxygen to tissues of the body. They circulate for about 120 days before they are engulfed by macrophages and recycled (Figure 1) [5, 7]. 

Erythrocytes can be derived *in vitro* from a variety of primary stem cell sources including umbilical cord blood (CB), peripheral blood (PB), and bone marrow (BM) [8, 9]. Despite their utility, these primary cells still represent donor-limited sources of blood substitutes. Human embryonic stem cells (hESCs) represent an alternative stem cell source for generating RBCs, one whose capacity for *in vitro* expansion far exceeds that of BM, PB, or even CB. Two different *in vitro *differentiation methods have been widely used to generate RBCs from hESCs: (1) embryoid body (EB) formation whereby hESCs are initially allowed to cluster and form three-dimensional spheres prior to creating single cell suspensions or (2) coculturing hESCs on top of animal stromal feeder cell layers. For example, Chang et al. successfully used an EB method to generate erythroid cells from hESCs. The resulting RBCs still had not enucleated after 30–56 days in culture and mainly expressed embryonic *ε*- and fetal *γ*-globins instead of the desired adult *β*-globin [10]. In another study, Olivier et al. used sequential stroma coculture steps to produce hESC-derived erythrocytes. In the first step, FH-B-hTERT stroma (human fetal liver cells immortalized with the catalytic subunit of telomerase reverse transcriptase) was used to induce initial differentiation of hESCs towards the hematopoietic lineage while MS5 cells (murine BM stroma cells) were used to further induce their differentiation towards erythrocytes. Despite their careful multistep approach and large yields (0.5 to 5 × 10^7^ cells), the resulting cells had similar problems to those generated by the EB method; they mainly expressed embryonic *ε* and fetal *γ* globin isoforms, with only a limited amount of adult *β*-globin being detectable [11]. 

Our recently developed “hemangioblast” system offers a clinically adaptable alternative to the above two methods and has proven to be useful for the large-scale generation of RBCs from hESCs [12–14]. Hemangioblasts (HBs) serve as the common precursor to both hematopoietic and endothelial cell lineages and therefore would be slightly upstream of HSCs in the hematopoietic hierarchy depicted in Figure 1. We found that hESCs can differentiate into HBs using a serum-free methylcellulose-based medium. Going through an intermediate HB stage prior to further differentiation enables a large expansion of multipotent cells and facilitates large-scale production of mature cell populations further downstream. We generated approximately 10^10^ to 10^11^ erythroid cells per six-well plate of hESCs using this system [14], which is over a thousandfold more efficient than the method reported above by Olivier et al. [11]. Extended *in vitro* culture on OP9 stromal cells facilitated enucleation in up to 65% of our cells and increased the expression of adult *β*-globin, allowing it to occur in up to 15% of the cells. Despite this improvement, the majority of cells were still found to express fetal and embryonic globin chains. 

Several groups, including our own, have demonstrated that human iPSCs can be used to generate erythrocytes [15–18]; however, our study also revealed that virus-transduced iPSCs contain intrinsic molecular and cellular abnormalities that may hinder their clinical applicability [18]. An alternative approach for RBC generation that bypasses the need for pluripotent stem cells altogether has recently been described [19]. Szabo et al. used ectopic Oct4 to transdifferentiate fibroblasts directly to CD45^+^ hematopoietic progenitors and by exposing them to EPO were able to produce erythroid lineage cells that expressed high levels of adult *β*-globin and low levels of fetal *ε*-globin and were capable of enucleation [19]. Further investigation will be required to determine which starting cell source, hESCs, iPSCs, or Oct4-transduced fibroblasts, will be the most useful for the development of *in vitro* generated RBC substitutes.

## 3. Platelets

Platelets (thrombocytes) play a central role in hemostasis (the stoppage of blood loss at sites of vascular injury) and vascular repair. Their concentration of ~3 × 10^11^/L makes them the second most abundant cell type in the peripheral blood, behind only RBCs (Figure 2) [4]. Platelets have a rather short lifespan compared to RBCs though and last only ~9 days in the circulation [5]. A serious condition called thrombocytopenia (platelet counts are <5 × 10^10^/L) can occur if platelet production is somehow defective as in patients with liver failure or leukemia, or if platelets are being destroyed, as in patients undergoing chemotherapy [20]. Platelet transfusions can be given to those suffering from life-threatening thrombocytopenia, yet transfusable platelets are often in short supply due to high demand and limited shelf life [20]. The inadequacies of donor-dependent programs have caused scientists and clinicians to become increasingly interested in developing alternative sources for functional, transfusable platelets. 

With no nucleus or DNA, platelets are actually cell fragments, being generated through the shearing and fragmentation of large, multinucleate megakaryocyte (MK) precursors. MKs arise in the bone marrow and share a common precursor with RBCs, the MEP (Figure 1). Progressive commitment of MEPs to the megakaryocyte lineage is principally regulated by thrombopoietin (TPO) and involves an increase in expression of the cell surface markers CD41 (*α*IIb/*β*3 integrin, or glycoprotein GPIIb/IIIa) and components of the GPIb/V/IX surface complex. Megakaryopoiesis also involves a substantial increase in cell size (50 to 100 *μ*m in diameter) caused by the cytosolic accumulation of platelet-associated proteins like von Willebrand Factor (vWF) [21] and nuclear polyploidization, resulting in the accumulation of up to 128N in DNA content [21, 22]. Cellular processes on the polyploid MK body called “proplatelets” begin to appear, and their eventual fragmentation and release results in the generation of platelets (Figure 1). The mechanism of platelet generation from MKs (thrombopoiesis) is not completely understood but appears to be extremely efficient *in vivo*, with 2,000–11,000 platelets being produced per MK [23].

*In vitro* megakaryopoiesis and thrombopoiesis was first reported in 1995 using CD34^+^ HSCs as a starting cell source [24], and several other studies have confirmed that hematopoietic stem/progenitors isolated from PB, BM, and CB are capable of producing MKs and functional platelets using standard cell culture methods [25–27]. In an attempt to recapitulate the BM microenvironment and provide more natural growth conditions, novel three-dimensional culture systems have recently been developed [28]. In one of these systems, researchers used surgical grade woven polyester fabric to create a 3D matrix within wells. In an improved system, the same research group used inverted colloidal crystals and polyacrylamide hydrogel to create a highly porous, highly interconnected network of spherical cavities within a 3D bioreactor. CD34^+^ cells were found to expand and differentiate into MKs and platelets within both 3D systems. Despite bioengineering advances, the limited *in vitro* expansion capabilities of primary CD34^+^ cells make these cells unable to replace donation as the principle source of platelets. hESCs may therefore be a better starting cell population for large-scale *in vitro* production. 

The first study to report the *in vitro* production of MKs from hESCs was published in 2006 using an OP9 coculture method [29], yet the MKs produced rarely generated any proplatelet-like structures. Since then, Takayama et al. has reported the successful generation of both MKs and functional platelets from hESCs and, more recently, from iPSCs [30, 31]. They cocultured hESCs or iPSCs on C3H10T1/2 stromal cells for 14-15 days, handpicked saclike structures containing hematopoietic progenitors and replated single cell suspensions onto fresh stroma in medium containing TPO, stem cell factor (SCF), and heparin for 9–23 days. Polyploid, CD41a/CD42b double positive MKs, emerged from these cultures and produced platelets containing characteristic morphology, as assessed by electron micrography [30]. A variety of *in vitro* tests confirmed platelet functionality, and a laser-induced vascular injury model was used to show that their iPSC-derived platelets readily incorporate into newly developing thrombi *in vivo* [31]. 

**Figure 3:**
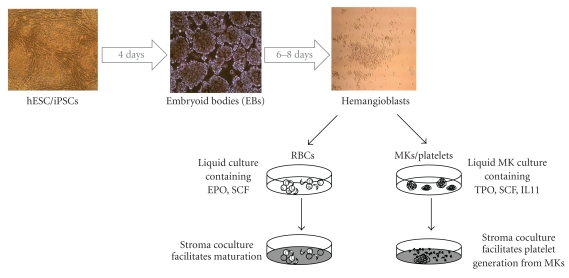
A hemangioblast (HB) differentiation system may be used to generate mature blood cells from hESCs/iPSCs. Pluripotent hESCs or iPSCs are first differentiated into EBs using a defined serum-free medium (Stemline II, Invitrogen) and vascular endothelial growth factor (VEGF), bone morphogenic protein 4 (BMP4), and basic fibroblast growth factor (bFGF). After 4 days, EBS are disrupted and single cell suspensions are replated into a serum-free, methylcellulose-based semisolid growth medium containing granulocyte colony-stimulating factor (G-CSF), GM-CSF, IL3, IL6, SCF, FL, VEGF, TPO, and bFGF for the generation of small, spherical, HBs (all images, 10x). After 6–8 days, HBs are harvested and grown in liquid culture containing the indicated cytokines in order to produce RBCs and platelets. For RBCs, subsequent coculture on stroma enhances enucleation and *β*-globin switching. For platelets, HBs are first differentiated into MKs in a stroma-free manner. Subsequent stroma coculture facilitates generation of functional platelets from the MKs.

The use of both serum and animal feeder layers throughout these studies hinders the ability of this method to be adapted for clinical use and handpicking ES sacs adds considerable time and labor to the process as well. Alternative methods will likely have to be developed for clinical grade, large-scale production. Towards this end, we have been able to use the HB system described above for RBC as an alternative, serum- and feeder-free method for the generation of MKs (Figure 3) [32]. Yet, similar to Takayama's studies, we also found that efficient platelet generation from MKs still requires conventional stroma coculture. Our hESC-derived platelets showed the ability to adhere to and spread on fibrinogen, vWF, and type I collagen-coated surfaces, to aggregate when stimulated with physiological agonists, and to retract fibrin clots *in vitro*. A laser-induced thrombosis model also confirmed that our hESC-platelets were capable of contributing to newly forming thrombi *in vivo* [32]. If *in vitro* differentiated hESCs are to become a major source of transfusable platelets, future work will be needed in order to determine a way to eliminate the need for stroma during the MK to platelet step and to increase the efficiency of *in vitro* thrombopoiesis as well.

## 4. White Blood Cells: Dendritic Cells

White blood cells (WBCs or leukocytes) only represent about 1% of the cells within the peripheral blood [4] (Figure 2), yet they play extremely important roles in protecting the body against viruses, bacteria, and the outgrowth of cancer cells. Straddling the interface between innate and adaptive immunity, dendritic cells (DCs) are one of the body's three main types of professional antigen-presenting cells (APCs). DCs can stimulate specific T-cell responses against a variety of disease-associated antigens and therefore, may be used in the development of vaccine-based therapies [33, 34].

Human DCs originate from HSCs and can develop through both myeloid and lymphoid lineage differentiation pathways [35]. Myeloid (m) DCs arise from granulocyte-monocyte progenitor- (GMP-) derived monocytes (Figure 1). They secrete interleukin (IL)12 in response to activating stimuli and express toll-like receptors TLR2 and TLR4. Lymphoid lineage-derived DCs (plasmacytoid (p) DCs) have similar functional characteristics to mDCs, but secrete interferon (IFN) *α*, and express TLR7 and 9 [35]. Immature DCs survive for weeks, sampling their surrounding environment in the skin, nose, lungs, gut, or peripheral blood and using TLRs for pattern recognition on various types of pathogens. Once in contact with a suitable antigen, immature DCs become activated and undergo the process of maturation, which involves proteolysing an antigen and presenting its fragments on the DC surface using MHC class I or II molecules. It also involves an upregulation in the expression of T-cell costimulatory receptors such as CD80 (B7.1), CD86 (B7.2) [36], and CD40 [37]. Maturing DCs also upregulate expression of CCR7, a chemotactic receptor that helps them migrate through the bloodstream to the spleen or into the lymphatic system [38]. Fully mature DCs only survive for a few days which is enough time for them to travel to the lymph nodes and activate helper T cells, killer T cells, and B cells. 

Innovative work performed in the late 1990s provided the proof of concept for clinical use of DCs as studies showed that *ex vivo* generated DCs (from allogenic or autologous BM or PB sources) could be loaded with melanoma-specific antigens and stimulate antitumor immune responses once injected into patients [39, 40]. Since then, other studies have shown that DCs exposed to killed tumor cells can also elicit specific cytotoxic CD8^+^ T-cell responses [41]. Given their powerful immunostimulatory effects, over 200 clinical trials are currently underway to explore the safety and efficacy of DC-based vaccines for diseases such as melanoma, multiple myeloma, type I diabetes, HIV, and hepatits C viral infections (http://www.clinicaltrials.gov/).

In April 2010, Provenge (Silpuleucil T, developed by Dendreon) became the first DC-based vaccine therapy to gain full FDA approval and is a treatment option for patients with metastatic castration-resistant prostate cancer [42]. This DC-based vaccine utilizes an antigenic peptide derived from prostatic acid phosphatase fused to the cytokine granulocyte macrophage colony-stimulating factor (GM-CSF) for highly efficient delivery and uptake by *ex vivo* cultured autologous DCs [43]. Given the high cost of tailor-made autologous or allogenic DC-based vaccines like Provenge [44], hESCs may serve as a cost-effective alternative cell source for the derivation and large-scale manufacture of antigen-primed DCs. 

Slukvin et al. were the first group to produce functional DCs from hESCs and did so by using a 3-step differentiation protocol adapted from the mouse ESC system [45]. They cocultured hESCs on OP9 stroma cells for 9-10 days to promote initial hematopoietic differentiation and then transferred cells to suspension culture consisting of *α*MEM, 10% fetal calf serum, and GM-CSF for the next 8–10 days. Live cells were purified and cultured in medium containing GM-CSF + IL4 for an additional 7–9 days, during which time human DCs emerged. Two other groups have since reported the generation of hESC-derived myeloid-lineage DCs using EB formation and have done so in a serum-free or serum- and feeder-free manner [46, 47]. These hESC-derived DCs had characteristic large eccentric nuclei, spiny dendritic processes and expressed DC surface markers CD11c, CD40, CD45, CD86, HLA class I, and HLA class II to varying degrees. Yields ranged from 2 DCs per hESC in one study [46] to 3–5 DCs per hESCs in a more recent study [47]. Despite subtle differences compared to monocyte-derived DCs, hESC-derived DCs appear to be functional upon maturation in assays measuring IL12p70 secretion, chemotaxis, antigen-uptake and proteolysis, induction of T-cell proliferation, and stimulation of antigen-specific cytotoxic CD8^+^ T-cell responses [46, 47]. 

When developing hESC-based DC vaccines, maturation cocktails will need to be carefully chosen in order to elicit the desired response *in vivo*. For example, prostaglandin E2 has been shown to facilitate DC chemotaxis, yet it inhibits the ability of DCs to secrete IL12p70 [48, 49]. This distinction would have important consequences for DC-based therapies *in vivo* since IL12p70 is essential for driving CD4^+^ T cells towards a proinflammatory, antimicrobial Th1 response and away from the opposing anti-inflammatory Th2 response. Clinical application of hESC-derived DC vaccines will also depend upon their performance in preclinical animal studies. *In vivo*, preclinical testing of hESC-DCs has not yet been reported, but studies performed with antigen-loaded autologous or allogenic DCs should provide a useful guide.

## 5. WBCs: Natural Killer Cells

Human natural killer (NK) cells are generated from HSC-derived CLPs (Figure 1) and have a half-life of ~7–10 days in the PB [50]. Their concentration of 1 × 10^8^ cells/L comprises ~1-2% of WBCs, or 0.01-0.02% of all cells in the PB [4]. NK cells belong to the innate immune system and provide rapid, nonspecific responses against various microbial infections and contribute to tumor cell detection and elimination [50]. NK cells mount a protective response if they encounter a cell with insufficient MHC I expression, yet, to prevent inappropriate cell killing, the procedure for surveying MHC I expression is rather complex. In brief, if a cell lacks sufficient self-MHC I molecules, interplay between various activating and inhibitory signals helps NK cells mount an appropriate protective response, either cytokine release, natural cytotoxicity or antibody-dependent cellular cytotoxicity (ADCC) [51]. 

The two main populations of NK cells, immature and mature, are functionally distinct and can be discerned based upon expression of various cell surface markers. Immature NK cells have high cytokine production capacity and low cytotoxicity potential and are typically CD56^bright^/CD16^low^/KIR^low^/CD94^high^ [52]. Secretion of cytokines by immature NK cells activates macrophages and helps initiate a broad immunological response. In contrast, mature NK cells display low cytokine production capacity and high cytolytic potential and are CD56^dim^/CD16^high^/KIR^high^/CD94^low^ [52]. Their cytolytic functions depend upon the release of granzyme and perforin enzymes from internal granules, which in turn are responsible for lysing and inducing apoptosis in target cells. The transition from immature to mature NK cells is thought to arise in secondary lymphoid tissues, yet the vast majority of NK cells in the PB are the mature CD56^dim^ CD16^high^ cytolytic type [53]. 

Endogenous NK cells may not detect and eliminate cancer cells *in vivo*. In many patients, NK cell activity may be reduced or defective, while in others, cancer cells have developed mechanisms to evade NK cell detection (reviewed in [51]). Nonetheless, studies published in the 1980s by Rosenberg and colleagues showed that infusions of autologous lymphokine-activated killer (LAK) cells stimulated *ex vivo* with IL2 were able to shrink tumors in patients with a variety of different types of cancer [54, 55]. This groundbreaking work stimulated considerable interest in using NK cells clinically and a variety of approaches for harnessing their cytotoxic capabilities. While the details of these studies are reviewed elsewhere [51], *ex vivo* stimulation and infusion of autologous or allogenic NK cells has been used in experimental therapies for many different types of cancers, and alterations to clinical protocols have increased the success of this therapeutic approach. Currently, over 200 clinical trials are being performed to evaluate the safety and efficacy of NK cell-based immunotherapy for leukemia, lymphoma, melanoma, glioma, renal cell carcinoma, and cancers of the breast, pancreas, lungs, head, and neck (http://www.clinicaltrials.gov/). Results of these trials will help establish the most effective strategies for harnessing NK immunotherapeutic potential. The biggest hindrance to these adoptive transfer approaches thus far appears to be the difficulty in obtaining sufficient numbers of NK cells from peripheral blood mononuclear or LAK cell collections [51]. The use of hESCs for *in vitro* generation of NK cells may provide larger pools of suitable effector cells and thus be able to overcome this hurdle.

In general, the differentiation of hESCs into lymphoid lineage cells has proven to be more difficult than their differentiation into myeloid lineage cells. Only one group, led by Dan Kaufman at the University of Minnesota, has been able to successfully and reproducibly derive functional NK cells from hESCs [56, 57]. Their optimized 2-step differentiation procedure begins by coculturing undifferentiated hESCs on M210-B4 (a mouse BM-derived stroma cell line that expresses laminin and collagen IV) for 17–20 days. CD34^+^/CD45^+^ double positive cells, which represent <5% of all cells, are then isolated from the culture and transferred onto AFT024 stroma in medium containing SCF, flt3-ligand (FL), IL7, and IL15. A highly enriched homogenous population of CD45^+^CD56^+^CD94^+^ NK cells typically emerges after 30–35 days. *In vitro *assays showed that hESC-derived NK cells produced in this manner were capable of secreting IFN*γ* in response to IL12/IL18 stimulation and also displayed potent natural cytotoxicity against K562 erythroleukemia cells and ADCC against Raji cells [56]. These hESC-NK cells were subsequently shown to harbor natural cytotoxicity against other types of cancer cells and displayed *in vivo* antitumor activity in a xenograft mouse model [57]. More recently, this same group has been able to successfully produce functional NK cells from iPSCs and showed that they harbor anti-HIV activity [58]. Despite these exciting findings, the requirement for two different types of stroma coculture as well as the need to isolate rare CD34/CD45 double positive cells limits the utility of this approach for large-scale, cost-effective, clinical grade production of hESC/iPSC-generated NK cells. Further optimization will need to be performed before such hESC/iPSC-derived NK therapies can move into clinical trials.

## 6. WBCs: T Cells

As part of the adaptive immune system, T cells develop in the thymus and can be stimulated to mount antigen-specific immune responses against a variety of pathogens and cancer cells. They are present at a concentration of ~1 × 10^9^ cells/L of peripheral blood, thus representing ~10% of WBCs or 0.1% of all circulating blood cells (Figure 2) [4]. While a detailed background on T-cell biology is beyond the scope of this paper, T cells can generally be divided into five main subtypes based on function and cell surface marker expression: effector or memory helper CD4^+^ T cells [59]; effector or memory cytotoxic CD8^+^ T cells [59]; immunosuppressive regulatory T cells (Tregs) [60]; skin, gut, or lung-resident *γδ* T cells [61]; rare, CD1d-restricted NK T cells [62]. 

Clinical interest in T cells as therapeutic agents largely revolves around the isolation and *ex vivo* expansion of specific antigen-responsive helper and/or cytotoxic T-cell subsets in order to generate a highly specific immune response once infused into a patient. CD4^+^ helper T cells will secrete particular cytokines in response to MHC II-presented antigens while CD8^+^ cytotoxic T cells will respond to MHC I-presented antigens and unload cytotoxic enzymes to induce apoptosis in antigen-expressing target cells. Adoptive T-cell therapy (ACT) was first described in 1988 as a treatment option for melanoma [63], and many improvements have been and are still being made to increase the utility, safety, and efficacy of ACT protocols [64–66]. For example, hundreds of tumor-associated antigenic peptides have been cloned [66] and enable the use of peptide-pulsed APCs to stimulate T cells *in vitro *prior to their use in patients. This approach has successfully been used to produce antigen-specific T cells for treating melanoma, HIV, leukemia, and other diseases [67–69]. Another strategy that is being developed to improve ACT protocols involves the use of genetic engineering to clone and artificially express antigen-specific T-cell receptors (TCRs) in autologous T-cell populations. This approach has been used successfully to treat melanoma [70, 71] and is being developed for use in HIV and leukemia therapies [72, 73]. 

The risks and complexities involved in exploiting the adaptive immune system make T-cell-based therapies incredibly expensive and still largely experimental. However, as exemplified above, the power of ACT is large enough to warrant efforts that might be able to streamline the therapy or make it more cost effective. Towards this end, several groups have devised protocols to differentiate T cells from pluripotent human cell sources. 

Extrapolating from results in various mouse studies, the use of immobilized Notch ligand, such as delta-1 (DL1), has proven to be an effective strategy for lymphopoietic differentiation of human pluripotent cells [74–76]. For example, Awong et al. showed that DL1-expressing OP9 stroma allowed CD34^+^ CB cells to differentiate into CD7^+^ T-cell progenitors. These pro-T cells could engraft into the thymuses of NOD/SCID/*γ*c^−/−^ (NSG) mice and continue differentiating down the T-cell lineage [75]. 

The first report that hESCs could be differentiated into T cells was published in 2006 [77]. Galić et al. cultured H1 hESCs on regular OP9 cells for 7–14 days, whereupon CD34^+^ cells were isolated and injected into a Thy/Liv implant within SCID or RAG2^−/−^ mice. The hESC-derived cells were found to differentiate into T cells within the thymus-like Thy/Liv environment [77]. Galić et al. switched to an EB-based method three years later and noted improvements in the Thy/Liv-generated, hESC-derived T cells [78]. CD4^+^/CD8^+^ double positive cells began to appear within 4 weeks while CD4^+^ single positive and CD8^+^ single positive cells that had undergone TCR rearrangements emerged within 8 weeks [78]. 

Another study published in 2009 showed that mature T cells could be obtained from hESCs using a completely *in vitro* culture system [79]. Timmermans et al. cocultured H1 hESCs on OP9 cells in *α*MEM plus 20% fetal calf serum for 12 days and observed the appearance of “hematopoietic zones,” which appear to be similar to the ES sacs described for megakaryocyte generation. They isolated CD34^hi^CD43^lo^ cells from these zones and replated them onto DL1-expressing OP9 cells for 5 to 7 weeks in the presence of FL, SCF, and IL7. CD45^+^CD7^+^CD117^+^cyCD3e^+^ T-cell progenitors emerged within 6 days while CD4^+^/CD8^+^ double positive cells emerged from a larger CD3e^+^CD5^+^ population within 14 days. After 30 days, CD3^+^ T cells that had undergone TCR rearrangements were present and found to be functional in assays examining their proliferation in response to phytohemagglutinin and IFN*γ* production [79]. In total, these differentiation protocols for T-cell generation have the potential to be further developed for use with iPSCs, and one day will hopefully be applicable to ACT protocols for human immunotherapy.

## 7. Other WBCs: Granulocytes and B Cells

Other leukocyte populations, including granulocytes (neutrophils, eosinophils, basophils) and B cells, may have utility as hESC-based therapies; however, interest in and/or development of these cell populations has not been as great as for other blood cell components. *In vitro* differentiation of hESCs down the B-cell lineage has been demonstrated in theory [80], yet detailed work is still needed in order to optimize differentiation conditions and functionally characterize the resulting cells. For granulocytes, the expense and difficulty in bringing hESC-based therapies to the clinic may not be warranted. For example, neutrophils are chemotactic phagocytes that migrate to sites of infection and provide protection against bacteria. Neutropenia (neutrophil counts less than 5 × 10^8^ cells/L) can cause an afflicted individual to be at higher risk for developing infections. While allogenic neutrophil transfusions were shown to alleviate the risk of infections over thirty years ago, the use of antibiotic, antiviral, and/or antifungal therapies has largely replaced them in the clinic [81].

Nonetheless, two studies published in 2009 describe the use of an EB-based method in order to generate CD11b^+^ neutrophils from KhES hESCs [82, 83]. These hESC-derived neutrophils expressed varying levels of other neutrophil cell surface markers and were slightly larger than those from the peripheral blood [83]. Despite such subtle differences, they were found to be functional in three *in vitro* assays evaluating chemotaxis, phagocytosis, and production of reactive oxygen species [82, 83]. One study also showed *in vivo* chemotaxis of hESC-derived neutrophils in response to IL1*β* expressed in an air-pouch inflammatory mouse model [82]. It remains to be determined whether or not hESC-neutrophils (or other types of granulocytes) will ever be developed for use as transfusion reagents, but *in vitro* differentiation systems for their generation may be useful for delineating cytokine requirements for hematopoietic differentiation, drug screening efforts, or elucidating molecular details of certain inherited diseases.

## 8. Concluding Remarks and Perspectives

Peripheral blood components have many different therapeutic applications, and hESCs have garnered a lot of interest as a renewable cell source that can be used for their generation. From RBCs and platelets being used in transfusions to treat cytopenias to DCs, NK cells, and T cells being used in immunotherapies to treat cancer and HIV, hESCs may be useful for generating these mature cell types in abundant supplies and in cost-effective ways (Table 1). Furthermore, iPSCs may be able to generate these mature cell types from a patient's own cells, thus reducing immunological barriers that plague cell-based therapies. 

The field of regenerative medicine is still in its infancy, yet some hESC-based therapies are starting to be tested in clinical trials. As of late 2010, two hESC-based therapies have been granted Investigational New Drug (IND) status by the FDA and have just recently entered (or will soon be entering) phase I/II clinical trials. Geron's GRNOPC1, consisting of hESC-derived oligodendrocyte progenitor cells, is being tested in clinical trials for treating subacute thoracic spinal cord injuries (http://www.geron.com/). Advanced Cell Technology's hESC-derived retinal pigment epithelium (RPE) cells (http://www.advancedcell.com/) will soon be tested in 2 clinical trials. The first trial is for the treatment of advanced Stargardt's Macular Dystrophy, a form of juvenile macular degeneration that often leads to blindness, and the second trial is for dry age-related macular degeneration. The safety and efficacy of these therapies in early clinical trials will likely have a significant impact on the development of other types of hESC-based therapies as well as the policies of the FDA towards the use of any hESC-derivatives to treat human disease.

## Figures and Tables

**Table 1:** Utility and current status of hESC/iPSC-derived blood components.

Cell type	Therapeutic use	Differentiation method	Advantages	Disadvantages
Erythrocytes (RBCs)	Transfusions for severe anemia or blood loss	EBs, HBs, and/or stroma coculture	Potential for alleviating shortages; production of pathogen-free (O)Rh^−^ “universal donor” RBCs	Inefficient enucleation; difficulties in switching to adult-type (beta) globin forms
Platelets	Transfusions for critical thrombocytopenia	Handpicking ES sacs with 2-step stroma coculture or HB method with 1-step stroma coculture	Potential for alleviating supply shortages due to high demand and limited shelf-life	Reliance on stroma and inefficiency/poor yield in MK to platelet differentiation step
Dendritic cells	Antigen-specific vaccines for cancer or HIV	EBs, serum- and stroma-free culture conditions	Cost-effective off-the-shelf potential; stimulates antigen- specific T-cell response	Animal models needed to test *in vivo* efficacy; may cause undesired side effects
Natural killer cells	Natural or antibody-assisted anticancer cytotoxicity	EBs with 2-step stroma-coculture and sorting of rare CD34^+^/CD45^+^ cells	Animal models suggest hES-derived NKs are highly effective	Reliance on 2 steps of stroma coculture; need for sorting may hinder clinical scaleup
T cells	antigen-specific anticancer or anti-HIV adoptive cell transfer	handpicking hematopoietic zones and 2-step stroma coculture including delta ligand expression	Cost-effective off-the-shelf therapeutic potential	Not efficient, needs further study; complex biology and high *in vivo* risks
